# Editorial: Metallodrugs in cancer therapy: past, present and new strategies

**DOI:** 10.3389/fchem.2024.1428502

**Published:** 2024-05-31

**Authors:** Irena Kostova

**Affiliations:** Department of Chemistry, Faculty of Pharmacy, Medical University-Sofia, Sofia, Bulgaria

**Keywords:** cancer, diagnostics, therapy, metal-based, pharmaceuticals

Cancer diagnostics and therapy that include metal-based pharmaceuticals have improved over the past decades. The therapeutic importance and application of metals and their compounds in medicine has been the subject of many investigations. Thus far, extensive research has been conducted to investigate the potential of many metal-based compounds and various protective and therapeutic drugs have been reported. Because of their specific mechanism of action and different pharmacological profile, these pharmaceuticals provide new perspectives for scientific research and for the development of antitumor drug design. Nonetheless, many challenges remain for the progress and application of metal-based anticancer therapeutics. There is a great necessity for metal-based drugs that are biodegradable, eliminable, and low toxic. All this requires more systematic studies on their biocompatibility and safety and warrants further investigations. That is why this field of research deserves more attention.

Metals in their cationic forms play vital roles in many essential biological functions and processes. The electron deficient metal cations easily interact with electron-rich biological molecules (proteins, DNA, etc.). Furthermore, metal cations have an affinity for various small biologically active molecules. The coordination of bioorganic compounds with metal cations can cause radical changes in the biochemical properties of both the metal cations and the ligand moieties that are known to have a broad variety of bioactivities.

In the last decades, quite a lot of classes of innovative metal-based organometallic and coordination complexes have been intensively explored as potential anticancer agents based on a wide variety of metals. Many drug candidates have shown most efficient antiproliferative and cytotoxic activity, with superior selectivity between malignant and normal cells, compared to traditional drugs. Despite the overabundance of recently designed metal complexes, their precise mechanisms of antineoplastic activity are often still unidentified. Apart from platinum group metals, there are many other metals in the periodic table with therapeutic potential. This delay in the therapeutic achievement of other metal-based antitumor agents has hindered progress in this area of research. The growing number of anticancer candidates among these metals proves this field offers a remarkable variety of new opportunities for the development of advanced unexplored pharmaceuticals with dissimilar and specific modes of action.

In line with this, the aim of the thematic Research Topic *“Metallodrugs in cancer therapy: past, present and new strategies”* was to highlight up-to-date evidence, forthcoming insights and recent advances in various parts of this broad research area. Review articles and research papers in the field have been considered for publication, providing standpoints and updates on the recent discovery of novel molecular targets as well as new treatments and medications with state-of-the-art mechanisms. Several excellent reviews on some of these agents, and some results of the clinical studies being performed, are discussed in detail.

The review of Moloudi et al. has been focused on cell cycle arrest (CCA) mechanism in apoptosis induced by photodynamic therapy (PDT). This review highlights the prospects to develop standardization of photodynamic therapy protocol and possibilities for combinative treatments. Two mathematical models of reactive oxygen species (ROS) level and their effects on oxidative stress have been introduced. Additionally, the mitotic checkpoints, delaying or arresting the cell cycle progression have been discussed. New strategies have been suggested, such as the combination of PDT with immunotherapy, radiation therapy, chemotherapy because of their synergistic effects for the efficient treatment. In addition to the known types of cell death (apoptosis, autophagy, necrosis), numerous alternative forms of cell death after PDT have been investigated (mitotic catastrophe, pyroptosis, paraptosis, necroptosis, ferroptosis, parthanatos). The review is focused on CCA induced by PDT for various photosensitizer agents on many cell lines. It gives new insights for the development of novel therapies. The authors have collected current *in vitro* and *in vivo* studies about cell cycle arrest post-PDT at different specific stages. For the standardization of PDT protocol, the authors have suggested several requirements, such as predicting tumor sensitivity, the choice of appropriate photosensitizer agents with low toxic effects and strong activity, the evaluation of ROS levels in the tumors, the immune system control, etc.

The review paper of Xu et al. provides a wide-ranging examination of the current knowledge of metal-based drugs in non-small cell lung cancer (NSCLC) treatment within the period (2010–2023). The authors have critically evaluated the known preclinical study methodologies, along with the respective controversies and inconsistencies. The most promising metal-based candidates have been highlighted and the disagreements and challenges in the current knowledge have been identified. Metal complexes of platinum, gold, ruthenium, and other metals, such as biogenic copper, iron, etc. metals have been discussed as promising candidates. Unlike natural compounds and other traditional organic therapeutics, the specificity of metal-based drugs is dominant as a result of their unique chemical properties, redox potentials, different coordination modes, specific molecular pathways and possible side effects. Their limitations (toxic effects, resistance, etc.), mechanisms, synergistic combinations have also been analyzed. Various mechanisms including DNA damage, redox modulation, and immunomodulation have been discussed. Artificial intelligence (AI) and machine learning (ML) revolutionize the research to identify the relationship between the properties and therapeutic consequences, thus helping the design of metal-based agents for optimum efficiency and protection. It has been concluded that metallodrugs offer promising opportunities and challenges in the treatment of NSCLC. The future directions, including optimization of the synthesis, clarification of resistance mechanisms, biomarkers, combination therapy, have been discussed.

The key antitumor drug Doxorubicin is broadly used in medical practice, in spite of its side effects, mainly cardiotoxicity. In order to inhibit doxorubicin-induced cardiotoxicity (DIC) many strategies have been investigated. It has been found that some endogenous metal cations can alleviate the side effects of the drug and delay cancer growth being part of DIC mechanism. Metal cations, for instance copper, iron, zinc, calcium have been recognized as important contributors to DIC. Zhou et al. have discussed the beneficial roles of these endogenous cations in the mitigation of cardiotoxicity, oxidative stress, DNA damage, mitochondrial dysfunction and other pathological mechanisms. Copper, iron, zinc and calcium exist in the human body in small quantities, but when absent, certain pathologies are possible. The overload and deposition of the metal cations can be regulated by specific metal chelators which cross easily the blood-brain barrier and do not affect the normal metabolic functions. Numerous drugs have been revealed to combat DIC, including chemical and natural compounds as well as traditional Chinese medicines, such as empagliflozin, sulforaphane, dexmedetomidine, etc. In the group of natural agents, puerarin, isoquercitrin, icariside II and many traditional Chinese medicine formulations are the most effective for maintaining the intracellular metal balance, especially for inhibiting ferroptosis. Additionally, Ginkgolide B, vitamins B and D, Epalastat analogue NARI-29, d-Limonene in combination with cyclodextrin can inhibit DIC via Ca-related directions. The anomalous zinc state during DIC can also be improved by using cannabidiol, Taurine zinc, zinc-curcumin, magnolol and other supplementations, thus playing a protective role. In the same way, free uncombined with proteins Cu is toxic and can cause atherosclerosis. Therefore, doxorubicin which promotes apoptosis should be administered with metal cation chelators which exhibit protective effects. Additional factors that impact the mechanism of DIC and different approaches for preventing and reducing the Doxorubicin cardiotoxicity have also been discussed. DIC can be improved by triggering classical signaling pathways, for instance ADAR2 overexpression, cAMP/PKA/SIRT1 or AKT/SIRT3/SOD2. Another DIC targets with cardioprotective role have also been analyzed, such as PDE10A, Sestrin 2, FAM134B, TFEB, etc. They reduce apoptosis, oxidative stress and endoplasmic reticulum stress, promote autophagy and improve cardiac function. Certain non-coding RNAs, such as (miR-128-3p, miR-451, miR-152, miR-125b, etc.), have shown evidence of reducing DIC. This comprehensive review discusses all these strategies, e.g., various metal ion chelators and natural products which can decrease ROS production, oxidative stress, mitochondrial dysfunction, and by this means to alleviate, prevent or treat the cardiotoxicity of Dox and ultimately to offer valuable insights for future research in this field.

The rational use of gold and its compounds in medical practice has a long history because of their strong therapeutic potential and lower adverse effects and cell resistance. In biological systems, the most preferred ligands for gold cations are the ligands which contain P- and S-donor atoms. Among the gold(I)-based drugs, the oral Au(I) compound auranofin (tetraacetyl-β-D-thioglucose-gold(I)-thioethylphosphine) has found wide application in medical practice. Originally developed for the rheumatoid arthritis treatment, Auranofin is currently under extensive investigations for oncological applications with great potential. Clinical trials with auranofin for ovarian and lung cancer therapy are in progress but its approval remains undecided. Like other Au(I) coordination complexes, auranofin is a monomeric neutral complex with a linear geometry. Triethylphosphine Au(I) cation is probably the principal auranofin metabolite which efficiently affect mitochondria inhibiting tumor growth under physiological conditions. Ding et al. have proved that some bi-gold compounds, such as BGC2a, were found more effective than the monomeric Au(I) drug auranofin owing to the increased Au(I) ion number that leading to advanced accumulation in mitochondria and its targeting and inhibition. The authors have synthesized and screened *in vitro* and *in vivo* several new bi-gold mitocans coordinated with different bi-phosphines and thiolate monosaccharides. The newly synthesized compounds have been found to display higher cytotoxicity with lower IC_50_ than the mono-gold agent auranofin and the bi-gold compound BGC2a in human lung cancer A549 and in lymphoma Jeko-1 cell lines. The most active gold compound of 1,3-bis(diphenylphosphaneyl)propane ligand, C3P4, accumulated in mitochondria, has strongly induced apoptosis and increased the total ROS and mitochondrial superoxide level. This compound significantly inhibited oxygen consumption rate and ATP production and should be further estimated as a promising agent for cancer therapy. The present study has shown that the increased number of gold cations and the thiolated monosaccharides and phosphines had strong impact on pharmacological activity.

The ongoing endeavors of researchers would certainly contribute to the progress and implementation of metallodrugs in cancer diagnosis and treatment. The Research Topic *“Metallodrugs in cancer therapy: past, present and new strategies”* provides the latest innovative studies conducted in this interdisciplinary field and proposes contemporary viewpoints on the multifaceted features of this quickly evolving fundamental and practical area, which is demonstrated by the wide variety of Research Topic and scientific approaches. All this can attract the consideration of scientists working in the anticancer drug discovery field In view of the ongoing challenges. This thematic Research Topic not only contributes to the knowledge and a substantial advance achieved in the field, but also provides new ideas and motivation for further new potential research directions and studies on potential therapeutic application of metal-based bioactive compounds for in cancer treatment.

